# Swine ANP32A Supports Avian Influenza Virus Polymerase

**DOI:** 10.1128/JVI.00132-20

**Published:** 2020-06-01

**Authors:** Thomas P. Peacock, Olivia C. Swann, Hamish A. Salvesen, Ecco Staller, P. Brian Leung, Daniel H. Goldhill, Hongbo Zhou, Simon G. Lillico, C. Bruce A. Whitelaw, Jason S. Long, Wendy S. Barclay

**Affiliations:** a Department of Infectious Diseases, Imperial College London, London, United Kingdom; b The Roslin Institute and Royal (Dick) School of Veterinary Studies, University of Edinburgh, Edinburgh, United Kingdom; c State Key Laboratory of Agricultural Microbiology, College of Veterinary Medicine, Huazhong Agricultural University, Wuhan, Hubei, People’s Republic of China; Cornell University

**Keywords:** ANP32, ANP32A, ANP32B, host factors, influenza, pandemic, swine, swine influenza, zoonotic, replication

## Abstract

Avian influenza viruses occasionally infect and adapt to mammals, including humans. Swine are often described as “mixing vessels,” being susceptible to both avian- and human-origin viruses, which allows the emergence of novel reassortants, such as the precursor to the 2009 H1N1 pandemic. ANP32 proteins are host factors that act as influenza virus polymerase cofactors. In this study, we describe how swine ANP32A, uniquely among the mammalian ANP32 proteins tested, supports the activity of avian-origin influenza virus polymerases and avian influenza virus replication. We further show that after the swine-origin influenza virus emerged in humans and caused the 2009 pandemic, it evolved polymerase gene mutations that enabled it to more efficiently use human ANP32 proteins. We map the enhanced proviral activity of swine ANP32A to a pair of amino acids, 106 and 156, in the leucine-rich repeat and central domains and show these mutations enhance binding to influenza virus trimeric polymerase. These findings help elucidate the molecular basis for the mixing vessel trait of swine and further our understanding of the evolution and ecology of viruses in this host.

**IMPORTANCE** Avian influenza viruses can jump from wild birds and poultry into mammalian species such as humans or swine, but they only continue to transmit if they accumulate mammalian adapting mutations. Pigs appear uniquely susceptible to both avian and human strains of influenza and are often described as virus “mixing vessels.” In this study, we describe how a host factor responsible for regulating virus replication, ANP32A, is different between swine and humans. Swine ANP32A allows a greater range of influenza viruses, specifically those from birds, to replicate. It does this by binding the virus polymerase more tightly than the human version of the protein. This work helps to explain the unique properties of swine as mixing vessels.

## Abstract (precis)

Avian influenza viruses can jump from wild birds and poultry into mammalian species such as humans or swine, but they only continue to transmit if they accumulate mammalian adapting mutations. Pigs appear uniquely susceptible to both avian and human strains of influenza and are often described as virus “mixing vessels.” In this study, we describe how a host factor responsible for regulating virus replication, ANP32A, is different between swine and humans. Swine ANP32A allows a greater range of influenza viruses, specifically those from birds, to replicate. It does this by binding the virus polymerase more tightly than the human version of the protein. This work helps to explain the unique properties of swine as mixing vessels.

## INTRODUCTION

Influenza A viruses continuously circulate in their natural reservoir of wild aquatic and sea birds. Occasionally, avian influenza viruses infect mammalian hosts, but these zoonotic viruses have to adapt for efficient replication and further transmission. This limits the emergence of novel endemic strains. Avian-origin, mammalian-adapted influenza viruses have been isolated from a range of mammalian species, including humans, swine, horses, dogs, seals, and bats (1–6).

One mammalian influenza host of significance are swine, which have been described as susceptible to viruses of both human and avian origin (6). It has been hypothesized that swine act as “mixing vessels,” allowing efficient gene transfer between avian- and mammalian-adapted viruses. This leads to reassortants, which are able to replicate in humans, but to which populations have no protective antibody responses, as best illustrated by the 2009 H1N1 pandemic (pH1N1) (7). The ability of pigs to act as mixing vessels has generally been attributed to the diversity of sialic acids, the receptors for influenza, found in pigs that would enable coinfection of a single host by diverse influenza strains (8, 9). The husbandry of swine has also been hypothesized to play a role in this mixing vessel trait; swine are often exposed to wild birds, and it is likely their environments are often contaminated with wild bird droppings containing avian influenza viruses (10, 11).

For an avian-origin influenza virus to efficiently infect and transmit between mammals, several host barriers must be overcome. One major barrier is the weak activity of avian influenza virus polymerases in the mammalian cell (12, 13). The acidic (leucine-rich) nuclear phosphoproteins of 32 kDa (ANP32) proteins are key host factors responsible for the restricted polymerase activity of avian influenza viruses in mammalian cells (14). ANP32 proteins possess an N-terminal domain composed of five leucine-rich repeats (LRRs) and a C-terminal low-complexity acidic region (LCAR) separated by a short region termed the “central domain.” In birds and most mammals, three ANP32 paralogues are found: ANP32A, ANP32B, and ANP32E (15, 16). The roles of ANP32 proteins in cells are diverse and often redundant between the family members but include histone chaperoning, transcriptional regulation, regulation of nuclear export, and apoptosis (16). In birds such as chickens and ducks, an exon duplication allows for the expression of an alternatively spliced, longer isoform of ANP32A that effectively supports activity of polymerases of avian influenza viruses (14, 17). Mammals only express the shorter forms of ANP32 proteins, which do not efficiently support avian polymerase unless the virus acquires adaptive mutations, particularly in the PB2 polymerase subunit, such as E627K (14). A further difference between the ANP32 proteins of different species is the level of redundancy in their ability to support influenza polymerase. In humans, two paralogues, ANP32A and ANP32B, are essential but redundant influenza polymerase cofactors (18, 19). In birds, only a single family member, ANP32A, supports influenza virus polymerase activity, as avian ANP32B proteins are not orthologous to mammalian ANP32B (15, 19, 20). In mice, only ANP32B can support influenza A polymerase activity (18, 19). Neither avian nor mammalian ANP32E proteins have been shown to support influenza polymerase activity (18–20).

In this study, we investigated the ability of a variety of mammalian ANP32 proteins to support influenza virus polymerases derived from viruses isolated from a range of hosts. We find differences in proviral efficiency that do not always coincide with the natural virus-host relationship; for example, human ANP32B is better able to support bat influenza polymerases than either bat ANP32 protein. Conversely, we describe evidence of human ANP32 adaptation early during the emergence of the pH1N1 virus from pigs, and find that swine ANP32A is the most potent proviral mammalian ANP32 protein tested, supporting nonadapted avian virus polymerase activity and avian influenza virus replication significantly better than human ANP32A. This can be attributed to amino acid differences in the LRR4 and central domains that enhance the interaction between swine ANP32A and the influenza polymerase complex, suggesting a mechanism for this enhanced proviral activity. Our findings give support to the special status as potential mixing vessels of swine in influenza evolution.

## RESULTS

### Mammals naturally susceptible to influenza have two proviral ANP32 proteins.

To investigate the ability of different mammalian ANP32A and ANP32B proteins to support influenza virus polymerase activity, several mammalian-origin influenza virus polymerase constellations were tested using an ANP32 reconstitution minigenome assay. A previously described human cell line with both ANP32A and ANP32B ablated (eHAP dKO) (18) was transfected with expression plasmids encoding ANP32A or ANP32B from chicken, human, swine, horse, dog, seal, or bat, as well as the minimal set of influenza polymerase expression plasmids for PB2, PB1, PA, and nucleoprotein (NP) to drive amplification and expression of a firefly luciferase viral-like reporter RNA and a *Renilla* luciferase expression plasmid as a transfection control.

**FIG 1:**
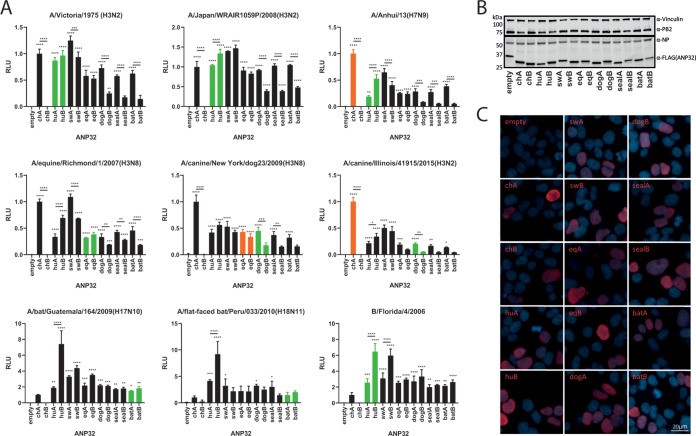
Most common mammalian influenza hosts have two ANP32 proteins capable of supporting influenza polymerase. (A) Minigenome assays performed in human eHAP dKO cells with ANP32 proteins from different avian or mammalian species cotransfected. Green bars indicate species from which the influenza virus polymerase was isolated; orange bars indicate recent species from which the virus has jumped. Data indicate triplicate repeats plotted as mean with standard deviation. Data for each polymerase normalized to chicken ANP32A. (B) Western blot assay showing protein expression levels of FLAG-tagged ANP32 proteins, NP, and PB2 during a minigenome assay. (C) Immunofluorescence images showing nuclear localization of all FLAG-tagged ANP32 proteins (red) tested. Nuclei are stained with DAPI (blue). ch, chicken; hu, human; sw, swine; eq, equine. Statistical significance was determined by one-way analysis of variance (ANOVA) with multiple comparisons against empty vector or between ANP32 proteins from the same host. *, 0.05 ≥ *P* > 0.01; **, 0.01 ≥ *P* > 0.001; ***, 0.001 ≥ *P* > 0.0001; ****, *P* ≤ 0.0001.

Initially, we tested a panel of polymerases derived from human, canine, equine, and bat influenza viruses. In contrast to chicken ANP32B, which does not support influenza virus polymerase activity (15, 19, 20), chicken ANP32A and all mammalian ANP32A and ANP32B proteins supported the activity of the mammalian-origin viral polymerases to various degrees (Fig. 1A). Among the mammalian ANP32 proteins tested, for most polymerases, swine ANP32A provided the strongest support of polymerase activity, whereas the ANP32B proteins from dog, seal, and bat displayed the least efficient proviral activity, lower than those species’ respective ANP32A proteins. These trends could not be explained by differences in expression levels or nuclear localization (Fig. 1B and C). The bat influenza polymerases, along with (human) influenza B polymerase, showed a different pattern of ANP32 usage, being able to strongly utilize ANP32Bs from all mammalian species, particularly human ANP32B (Fig. 1A). There was no evidence that influenza viruses adapted to particular mammals had evolved to specifically use the corresponding ANP32 proteins. For example, dog ANP32A or ANP32B were not the most efficient cofactors for canine influenza virus polymerase, and human ANP32B was better able to support the bat influenza polymerase than either of the bat ANP32 proteins.

### Swine ANP32A, but not other mammalian ANP32 proteins, can support the polymerase activity and virus replication of avian-origin influenza viruses.

**FIG 2:**
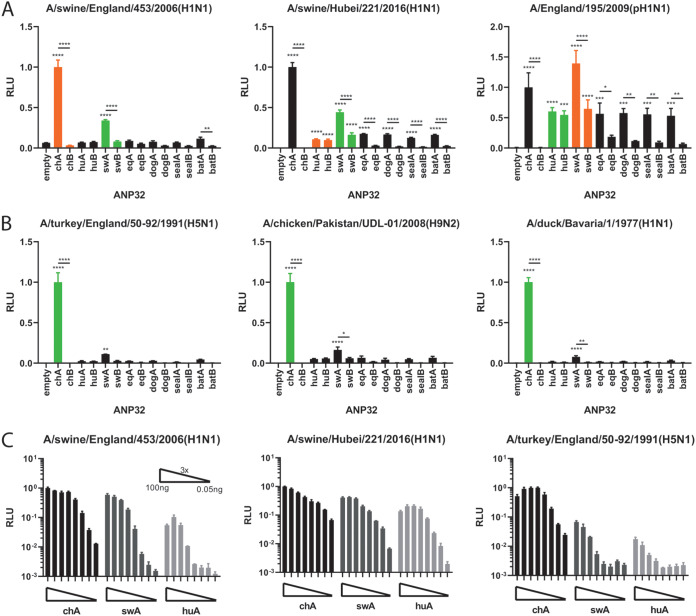
swANP32A can support the activity of minimally mammalian-adapted or completely nonadapted polymerases. Minigenome assays of swine (A) and avian (B) polymerases performed in human eHAP dKO cells with ANP32 proteins from different avian or mammalian species cotransfected. Green bars indicate species from which the influenza virus polymerase was isolated; orange bars indicate recent species from which the virus has jumped. Data indicate triplicate repeats plotted as mean with standard deviation. Data for each polymerase normalized to chicken ANP32A. (C) ANP32 protein titrations with three different virus polymerase constellations. ANP32 expression plasmids were diluted in a series of 3× dilutions starting with 100 ng. Data indicate triplicate repeats plotted as mean with standard deviation. Statistical significance was determined by one-way analysis of variance (ANOVA) with multiple comparisons against empty vector. **, 0.01 ≥ *P* > 0.001; ***, 0.001 ≥ *P* > 0.0001; ****, *P* ≤ 0.0001.

We next tested the ANP32 preference of a human 2009 (swine-origin) pH1N1 and two polymerases from swine influenza isolates. Interestingly, these polymerases were robustly supported by chicken and swine ANP32A but not other mammalian ANP32 proteins, with the Eurasian avian-like polymerase from A/swine/England/453/2006 (EAH1N1) (EAH1N1 sw/453) showing the clearest effect (Fig. 2A). We went on to test a panel of avian-origin viral polymerases with no known mammalian polymerase adaptations, including A/duck/Bavaria/77(H1N1) (H1N1 Bav), thought to be an avian precursor of the Eurasian avian-like swine lineage (Fig. 2B) (5). For all the avian origin viral polymerases, the stringent preference for avian ANP32A to support polymerase activity was evident (coexpression of chicken ANP32A led to very strong polymerase activity). However, among all the mammalian ANP32 proteins tested, only swine ANP32A was able to significantly support avian influenza polymerase activity, though to a lesser degree than chicken ANP32A (Fig. 2B). This unique proviral effect of swine ANP32A on swine and avian-origin polymerases was maintained across a wide titration of plasmid doses (Fig. 2C).

**FIG 3:**
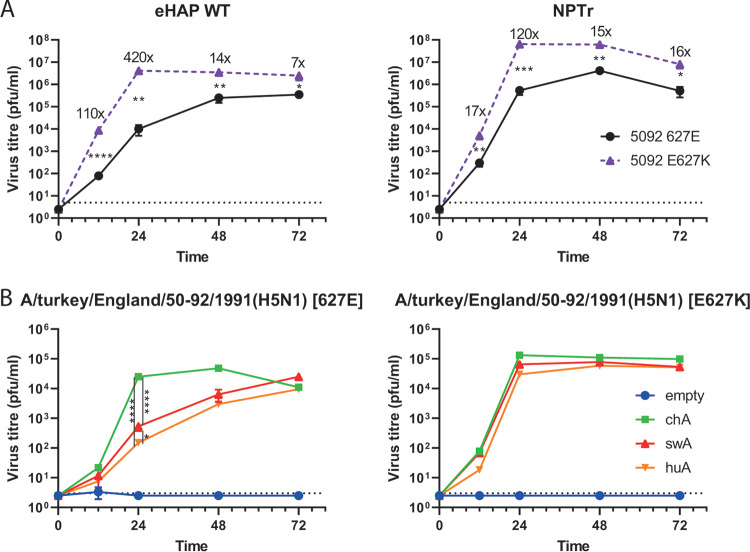
Swine ANP32A can support avian influenza virus replication better than human ANP32A. Comparative growth kinetics of isogenic, recombinant avian influenza viruses (A/turkey/England/50-92/1991 [H5N1]) PB2 627E (wild type) versus E627K in wild-type human eHAP cells and swine NPTr cells (A) and eHAP dKO cells (B) preexpressing empty vector, chicken, swine, or human ANP32A. Cells were infected at a multiplicity of infection (MOI) of 0.001. All time points taken in triplicate, and mean viral titers were determined by plaque assay in MDCK cells with the standard deviation shown. Graph shows representative data of at least two independent repeats showing the same trends. Statistical significance determined by multiple Student’s *t* tests in panel A and one-way analysis of variance (ANOVA) with multiple comparisons in panel B. Value shown on graph in panel A indicate fold change in mean titers. Dotted lines on graphs indicate limits of detection. *, 0.05 ≥ *P* > 0.01; **, 0.01 ≥ *P* > 0.001; ***, 0.001 ≥ *P* > 0.0001; ****, *P* ≤ 0.0001.

Furthermore, we tested the relative ability of human and swine cells to support the replication of a nonadapted avian influenza virus. Isogenic recombinant A/turkey/England/50-92/1991(H5N1) (H5N1 50-92) virus containing either wild-type PB2 (E627) or the mammalian adaptation PB2-E627K were used to infect wild-type human eHAP and swine NPTr cells (Fig. 3A). Although E627K significantly increased the virus replication in both cell lines, the magnitude of difference was less in the swine cells than the human cells at earlier time points (for example, 17-fold versus 110-fold at 12 h postinfection). The less drastic reduction in replication of the virus with nonadapted avian-origin polymerase compared with the adapted control in swine cells is consistent with the hypothesis that swine ANP32A can support the replication of avian influenza viruses.

To investigate whether this difference was indeed accounted for by differences in ANP32A proteins, chicken, swine, or human ANP32A were preexpressed in eHAP dKO cells that were then infected with 50-92 wild-type and E627K recombinant viruses (Fig. 3B). As shown previously, when the empty vector was expressed, no virus replication took place (18). For the mammalian-adapted PB2-E627K virus, it made little difference which ANP32A protein was expressed, although a trend was seen for chicken ANP32A supporting higher titers than swine ANP32A, which, in turn, supported higher titers than human ANP32A. For the nonadapted PB2-E627 virus, however, a greater and significant difference was seen—chicken ANP32A clearly supported virus replication better than either mammalian ANP32A protein. Swine ANP32A supported replication of the avian influenza virus to a higher level than human ANP32A at all time points, and this difference was significant (*P* < 0.05) at 24 h postinfection. Overall, this indicates that swine ANP32A is better able to both support avian influenza virus polymerase activity, as well as virus replication, than human ANP32A.

### The pH1N1 swine influenza virus polymerase, adapting to humans, evolved to better use human ANP32 proteins.

In 2009, the swine-origin pH1N1 influenza virus adapted from pigs for transmission between humans, causing an influenza pandemic (7). The pH1N1 polymerase genes were derived from a swine triple-reassortant constellation in which PB2 and PA originally derived from avian influenza viruses in the mid-1990s (21). From 2009 to 2010, the virus continued to circulate and adapt to humans in the second and third pandemic waves (22). pH1N1 viruses contain the PB2 polymerase adaptations T271A, G590S, and Q591R, which appear to compensate for the lack of E627K in enabling replication in mammalian cells, and these amino acids did not change between the first and third waves of the pandemic (23). We previously showed that a single substitution in the PA subunit of the polymerase, N321K, contributed to increased polymerase activity of third-wave pH1N1 viruses in human cells (22). We hypothesized that this PA mutation might function by improving support for the emerging virus polymerase by the human ANP32 proteins.

**FIG 4:**
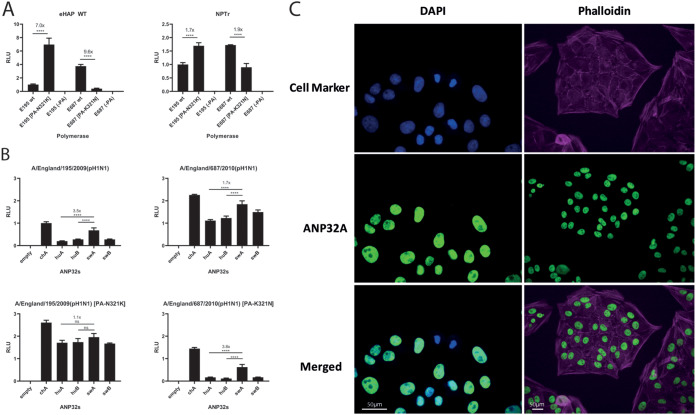
Third-wave pandemic H1N1 viruses adapt to human ANP32 proteins through the PA mutation N321K. (A) Minigenome assays of polymerases derived from first- and third-wave pH1N1 viruses (E195 and E687, respectively) performed in wild-type human eHAP cells and swine NPTr cells. Data indicate triplicate repeats plotted as mean with standard deviation. Data normalized to wild-type pH1N1 E195. (B) Minigenome assays performed in human eHAP cells with ANP32A and ANP32B knocked out and complemented with ANP32 proteins from human or swine following cotransfection of expression plasmids. Data indicate triplicate repeats plotted as mean with standard deviation. Data normalized to pH1N1 E195 wild type with chicken ANP32A. All experiments in panels A and B performed on two separate occasions with a representative repeat shown. (C) Indirect immunofluorescence images showing endogenous nuclear localization of swine ANP32A in swine NPTr cells. Statistical significance was determined by one-way analysis of variance (ANOVA) with multiple comparisons. ****, *P* ≤ 0.0001.

We performed minigenome assays with a first-wave pandemic virus, A/England/195/2009(pH1N1) (pH1N1 E195), and a third-wave pandemic virus, A/England/687/2010(pH1N1) (pH1N1 E687), which differ in PA at position 321. As shown before, PA 321K enhances polymerase activity in general in both virus polymerase backgrounds in human eHAP cells, as well as swine NPTr cells (Fig. 4A). However, the boost is far greater in the human cells (∼7-fold) than in the swine cells (∼2-fold), implying this mutation may have arisen to overcome the greater restriction seen upon the jump into humans (22).

We next tested the ability of human and swine ANP32 proteins to support the different pH1N1 polymerases in eHAP dKO cells. Polymerases containing PA-321N are more robustly enhanced by swine ANP32A (by around 3.5-fold compared to human ANP32A), as is typical of swine-origin polymerases (Fig. 4B). Swine ANP32A, however, gives a much more modest boost to polymerase activity than human ANP32A when 321K is present (<2-fold). This suggests the PA N321K adaptation was selected in these viruses to adapt to the more poorly supportive ANP32 proteins present in human cells. We could further show that endogenous swine ANP32A protein is predominantly localized in the nucleus in swine NPTr cells, consistent with our previous overexpression data (Fig. 4C).

### Differences in swine and human ANP32A proviral activity can be mapped to the LRR4 and central region.

**FIG 5:**
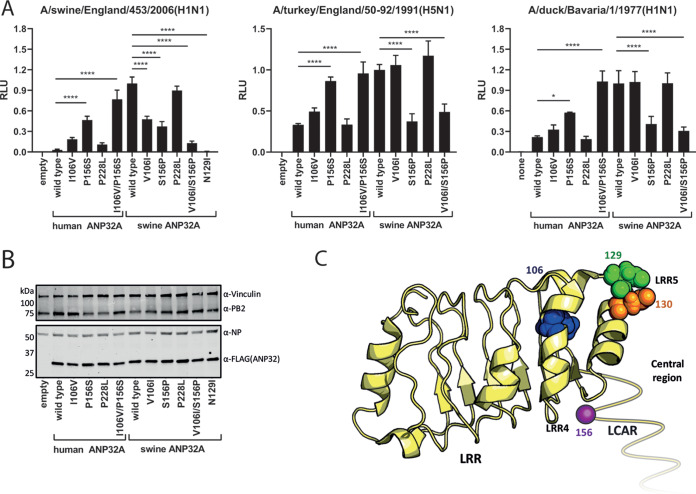
The enhanced proviral activity of swine ANP32A maps to amino acids in LRR4 and the central domain. (A) Minigenome assays with polymerase constellations from a swine or an avian influenza virus performed in human eHAP dKO cells with human/swine ANP32A reciprocal mutants expressed. Data indicate triplicate repeats plotted as mean with standard deviation repeated on two separate occasions with a representative repeat shown. Data normalized to each polymerase with swine wild-type ANP32A. (B) Western blot analysis showing expression levels of human/swine ANP32A from minigenome assays. (C) Crystal structure of ANP32A (PDB accession no. 2JE1) with residues found to affect proviral activity mapped (39). The unresolved, unstructured LCAR is shown as a yellow line. Schematic made using PyMol (40). Statistical significance was determined by one-way analysis of variance (ANOVA) with multiple comparisons. *, 0.05 ≥ *P* > 0.01; ***, 0.001 ≥ *P* > 0.0001; ****, *P* ≤ 0.0001.

We set out to identify the molecular basis for the unusually high activity of swine ANP32A in comparison with the other mammalian ANP32 proteins. An alignment of ANP32A primary sequences identified three amino acids outside the LCAR that differed between swine ANP32A and the other mammalian orthologues. Using reciprocal mutant ANP32A proteins, the identity of amino acid position 156, naturally a serine in swine ANP32A but a proline in most other mammalian and all avian ANP32A proteins, was shown to have a major reciprocal influence on activity (Fig. 5A). The amino acid at position 106 contributed to a lesser degree, with swinelike valine enhancing proviral activity over humanlike isoleucine when complementing the swine influenza polymerase constellation, though changes at this residue appeared to have more minor effects on proviral activity supporting the 50-92 and Bav avian virus polymerases. Position 228, localized near the C-terminal nuclear localization signal of ANP32A, had no appreciable impact. In the background of human ANP32A, I106V generally gave between a 1.5- and 6-fold increase in polymerase activity, while P156S gave between a 3- and 16-fold boost, depending on the polymerase constellation tested. The combined 106/156 mutant showed an additive effect, implying these two residues are, together, responsible for the enhanced proviral activity of swine ANP32A (Fig. 5A and C). None of the mutations affected expression levels (Fig. 5B). Positions 106 and 156 map to the LRR4 and central domains of ANP32 protein, respectively, proximal to the previously characterized LRR5 residues, 129 and 130, that are responsible for the lack of proviral activity of avian ANP32B proteins (Fig. 5C) (15, 19). This reinforces the concept that the LRR4/LRR5/central region of ANP32 proteins is essential to their proviral function. Indeed, we could show that introducing the mutation N129I into swine ANP32A abrogated its ability to support influenza polymerase activity (Fig. 5A).

### An increase in binding to the polymerase accounts for the enhanced proviral activity of swine ANP32A.

Proviral ANP32 proteins from birds and mammals directly bind trimeric polymerase in the cell nucleus (17, 24, 25). Moreover, the inability of avian ANP32B to support influenza polymerase activity correlates with a lack of protein interaction conferred by amino acid differences at residues 129 and 130 (15).

**FIG 6:**
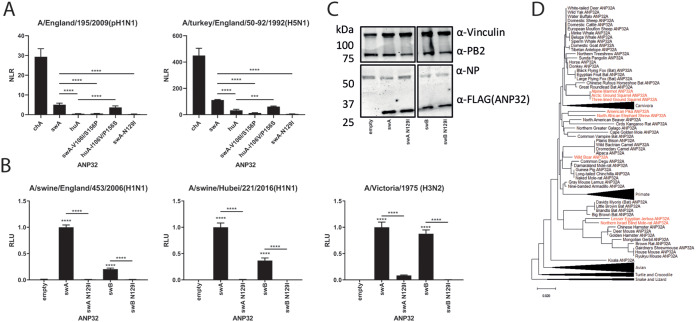
Amino acid residues responsible for the enhanced support of polymerase activity of swine ANP32A also mediate increased binding to influenza trimeric polymerase. (A) Split-luciferase assays showing the relative binding of different ANP32 proteins to trimeric polymerase from human pH1N1 or avian H5N1 viruses. PB1 was tagged with the N-terminal part of *Gaussia* luciferase, while ANP32 proteins were tagged with the C-terminal part. NLR, normalized luminescence ratio, calculated from the ratio between tagged and untagged ANP32/PB1 pairs. Assay performed in 293T cells. Data indicate triplicate repeats plotted as mean with standard deviation, repeated across two separate experiments with representative data shown. Statistical significance was determined by one-way ANOVA with multiple comparisons between the swA and huA wild types and mutants. ***, 0.001 ≥ *P* > 0.0001; ****, *P* ≤ 0.0001. (B) Minigenome assays with reconstituted polymerases from 3 different influenza viruses, performed in human eHAP cells with ANP32A and ANP32B knocked out and complemented with wild-type swine ANP32A or B or N129I mutants thereof. Data indicate triplicate repeats plotted as mean with standard deviation, repeated across two separate experiments with representative data shown. Data normalized to each polymerase with wild-type swine ANP32A. ****, *P* ≤ 0.0001. (C) Western blot assay showing protein expression levels of FLAG-tagged swine ANP32 wild type or N129I proteins during a minigenome assay. (D) Phylogenetic tree of mammalian ANP32A proteins. Species that contain the highly proviral 156S shown in red; species with 156P shown in black. Phylogenetic trees made using the neighbor-joining method based on amino acid sequence. Statistical significance was determined by one-way analysis of variance (ANOVA) with multiple comparisons against an empty vector.

To assess the strength of the interaction between swine ANP32A protein and influenza polymerase, we used a split-luciferase assay, where the two halves of *Gaussia* luciferase are fused onto PB1 and ANP32 proteins (15, 25). As seen previously (25), the interaction between influenza virus polymerase and human ANP32A was weak but detectable above the background (huA) (Fig. 6A). Swine ANP32A interacted more strongly with both human-origin E195 (pH1N1 2009) and avian-origin A/turkey/England/50-92/1991(H5N1) influenza polymerases, although not as strongly as chicken ANP32A (Fig. 6A). Furthermore, the two residues identified as being responsible for the strong proviral activity of swine ANP32A, at positions 106 and 156, enhanced polymerase binding by human ANP32A, and the reciprocal mutations decrease the swine ANP32A interaction, implying the mode of action of these mutations is through enhancing swine ANP32A-polymerase interactions (Fig. 6A). It was also shown that N129I, the substitution naturally identified in chicken ANP32B and previously shown to abolish binding and activity in chicken and human ANP32 proteins (15, 19), showed a similar phenotype in swine ANP32A, abolishing detectable binding and activity (Fig. 6A and B). The ablations of the proviral activity of swine ANP32A and ANP32B by the substitution N129I were not explained by reductions in expression of these mutant proteins (Fig. 6B and C).

### Estimating the proviral activity of ANP32A proteins from other mammalian species.

Based on the molecular markers described in this study, it is possible to survey ANP32A proteins from all mammals to predict which other species may have highly influenza polymerase-supportive proteins and therefore the potential to act as mixing vessels for reassortment between avian- and mammalian-adapted influenza viruses.

Very few mammals share the proviral marker 156S, and the few that do mostly constitute species not yet described as hosts for influenza viruses (Fig. 6D). A notable exception is the pika, which, in a similar manner to pigs, is known to often become infected with avian influenza viruses with minimal mammalian adaptation (26–28). Pigs are currently the only known mammalian species with a publicly available ANP32A sequence that contain the secondary, minor proviral maker 106V.

## DISCUSSION

In this study, we describe the ability of different mammalian ANP32A and ANP32B proteins to support the activity of influenza virus polymerases isolated from a variety of hosts. We found that swine ANP32A, uniquely among the ANP32 proteins, supports avian influenza virus polymerase activity and virus replication. Swine ANP32A does not harbor the avian-specific 33-amino acid duplication that enables the strong interaction and efficient support of polymerase activity of avian-origin viruses by avian ANP32A proteins (14). Thus, avian influenza viruses are restricted for replication in swine as we have previously shown, and mammalian-adapting mutations enhance their polymerase activity in pig cells (11). Nonetheless, this level of proviral activity associated with swine ANP32A, albeit weaker than avian ANP32As, may contribute to the role of swine as mixing vessels; nonadapted avian influenza viruses that infect pigs could replicate sufficiently to accumulate further mutations that allow for more efficient mammalian adaptation and/or reassortment, enabling the virus to either become endemic in swine or to jump into other mammals, including humans.

We map this strongly proviral polymerase phenotype to a pair of mutations which allow swine ANP32A to bind more strongly to influenza virus polymerase, potentially explaining the mechanism behind its enhanced proviral activity. These residues are only found in a few other mammals, including pika. It is conceivable these residues are located at a binding interface between polymerase and ANP32, but resolution of the structure of the host:virus complex will be required to confirm this hypothesis.

A recent study from Zhang and colleagues independently corroborated the superior ability of swine ANP32A among mammalian ANP32 proteins to support avian influenza virus polymerase activity (29). Moreover, they also correlated this phenotype with amino acids at positions 106 and 156 that increased the strength of interactions between the host factor and the viral polymerase complex. In their studies, the interaction between ANP32 proteins and viral polymerase was measured by coimmunoprecipitation, making it unlikely that the similar differences we measured using our quantitative split-luciferase assay were due to reorientation of the luciferase tags.

It has long been speculated that swine play a role as mixing vessels by acting as host to both human- and avian-origin influenza viruses (30). This trait may be partially attributed to receptor patterns in swine allowing viruses that bind to both α2,3-linked (i.e., avian-like viruses) and α2,6-linked sialic acid (i.e., humanlike) to replicate alongside each other (8, 9). However, replication of the avian-origin influenza virus genomes inside infected cells is also required to enhance the opportunity for further adaptation or reassortment. We previously developed a minigenome assay for assessing polymerase activity in swine cells and showed that avian virus polymerases were restricted and that restriction could be overcome by typical mutations known to adapt polymerase to human cells (11). Taken together, the ability to enter swine cells without receptor-switching changes in the hemagglutinin gene, along with a greater mutation landscape afforded in swine cells by the partially supportive proviral function of swine ANP32A, may have an additive effect to allow swine to act an intermediate host for influenza viruses to adapt to mammals. Furthermore, our work implies other mammals, such as the pika, could play a similar role, which is of particular interest due to the pika’s natural habitat often overlapping with that of wild birds and its (somewhat swinelike) distribution of both α2,3- and α2,6-linked sialic acid receptors (31).

Upon crossing into humans from swine, it is likely that viruses would be under selective pressure to adapt to human proviral factors, such as the ANP32 proteins. We use the example of a pair of first- and third-wave pandemic H1N1 influenza viruses isolated from clinical cases in 2009 and 2010 (22). The polymerase constellation of the 2009 pH1N1 virus contains PB2 and PA gene segments donated from avian sources to a swine virus in a triple-reassortant constellation in the mid-1990s, then passed onto humans in 2009 (21). Although the first-wave viruses, derived directly from swine, can clearly replicate and transmit between humans, over time, the PA substitution, N321K, was selected because it enabled more efficient activity of the viral polymerase in human cells. Our data suggest this is a direct adaptation to human ANP32 proteins. This again illustrates how swine have acted as a “halfway house” for the stepwise adaptation of genes originating in avian influenza viruses that have eventually become humanized.

Also of note, we show here that as for the human orthologues (18, 19), the ANP32A and B proteins of swine (as well as all other mammals tested here) are redundant in their ability to support the viral polymerase. We further show that the substitution N129I is able to partially or fully ablate the proviral activity of swine ANP32A and ANP32B. We suggest that the introduction of this substitution in both swine ANP32A and ANP32B by genome editing would be a feasible basis for generating influenza-resistant, or -resilient, pigs in a similar manner to that demonstrated for porcine respiratory and reproductive syndrome virus-resistant pigs, and proposed for influenza-resistant, or -resilient, chickens (15, 32).

To conclude, we hypothesize that the superior proviral function of swine ANP32A for supporting influenza replication may enable swine to act as intermediary hosts for avian influenza viruses and also affect the way the viruses evolve as they pass from birds through swine and into humans. This, in turn, may influence the ability of different swine influenza viruses to act as zoonotic agents or as potential pandemic viruses.

## MATERIALS AND METHODS

### Cells.

Human-engineered Haploid cells (eHAP; Horizon Discovery) and eHAP cells with ANP32A and ANP32B knocked out (dKO) by CRISPR-Cas9, as originally described in reference 18, were maintained in Iscove’s modified Dulbecco’s medium (IMDM; Thermo Fisher) supplemented with 10% fetal bovine serum (FBS; Biosera), 1% nonessential amino acids (NEAA; Gibco), and 1% penicillin-streptomycin (Pen-Strep) (Invitrogen). Human embryonic kidney (293Ts, ATCC), newborn pig trachea cells (NPTr; ATCC), and Madin-Darby canine kidney cells (MDCK; ATCC) were maintained in Dulbecco’s modified Eagle medium (DMEM) supplemented with 10% FBS, 1% NEAA, and 1% Pen-Strep. All cells were maintained at 37°C and 5% CO_2_.

### ANP32 plasmids constructs.

Animal ANP32 constructs were codon optimized and synthesized by GeneArt (Thermo Fisher). Sequences used were pig (Sus scrofa) ANP32B (GenPept accession no. XP_020922136.1), horse (Equus caballus) ANP32A (GenPept accession no. XP_001495860.2) and ANP32B (GenPept accession no. XP_023485491.1), dog (Canis lupus familiaris) ANP32A (GenPept accession no. NP_001003013.2), dingo (Canis lupus dingo) ANP32B (GenPept accession no. XP_025328134.1), monk seal (Neomonachus schauinslandi) ANP32A (GenPept accession no. XP_021549451.1) and ANP32B (GenPept accession no. XP_021546921.1), and common vampire bat (Desmodus rotundus) ANP32A (GenPept accession no. XP_024423449.1) and ANP32B (GenPept accession no. XP_024415874.1). All isoforms were chosen based on their orthology and synteny to the known functional human isoforms. Species of origin were chosen due to being influenza hosts or the most commonly related species to influenza hosts (in the case of monk seal, which is closely related to harbor seal, whereas common vampire bats belong to the same family as little yellow-shouldered and flat-faced bats). Dingo ANP32B was substituted for dog ANP32B, as the equivalent isoform used for all other ANP32Bs is unannotated in the dog genome due to a gap in the scaffold. All ANP32 expression constructs included a C-terminal GSG linker followed by a FLAG tag and a pair of stop codons. Overlap extension PCR was used to introduce mutations into the ANP32 constructs, which were then subcloned back into pCAGGs and confirmed by Sanger sequencing.

### Viral minigenome plasmid constructs.

Viruses and virus minigenome full strain names used through this study were A/Victoria/1975(H3N2) (H3N2 Victoria), A/England/195/2009(pH1N1) (pH1N1 E195), A/England/687/2010(pH1N1) (pH1N1 E687), A/Japan/WRAIR1059P/2008(H3N2) (H3N2 Japan), B/Florida/4/2006 (B/Florida), A/Anhui/2013(H7N9) (H7N9 Anhui), A/duck/Bavaria/1/1977(H1N1) (H1N1 Bavaria), A/turkey/England/50-92/1991(H5N1) (H5N1 50-92), A/chicken/Pakistan/UDL-01/2008(H9N2) (H9N2 UDL1/08), A/canine/New York/dog23/2009(H3N8) (CIV H3N8), A/canine/Illinois/41915/2015 (H3N2) (CIV H3N2), A/equine/Richmond/1/2007(H3N8) (H3N8 Richmond), A/swine/England/453/2006 (EAH1N1 sw/453), A/swine/Hubei/221/2016(H1N1) (H1N1 Hubei), A/little yellow-shouldered bat/Guatemala/164/2009(H17N10) (H17N10 H17), and A/flat-faced bat/Peru/033/2010(H18N11) (H18N11 H18). Viral minigenome expression plasmids (for PB2, PB1, PA, and NP) for H3N2 Victoria, H5N1 50-92, H1N1 E195, H1N1 E687, B/Florida, H9N2 UDL1/08, and H1N1 Bavaria have been previously described (11, 14, 22, 33). Viral minigenome plasmids for H1N1 sw/453, H3N2 Japan, CIV H3N2, CIV H3N8, H3N8 Hubei, and H3N8 Richmond were subcloned from reverse genetics plasmids or cDNA into pCAGGs expression vectors using virus segment-specific primers.

pCAGGs minigenome reporters for H17N10 and H18N11 bat influenza viruses were a kind gift from Martin Schwemmle, Universitätsklinikum Freiburg (34). pCAGGs minigenome reporters for H7N9 were a kind gift from Munir Iqbal, The Pirbright Institute, United Kingdom. Reverse genetics plasmids for H3N8 Richmond were a kind gift from Adam Rash of the Animal Health Trust, Newmarket, United Kingdom. Reverse genetics plasmids for H3N2 CIV and H3N8 CIV were a kind gift from Colin Parrish of the Baker Institute for Animal Health, Cornell University (35, 36). Viral RNA from sw/453 was kindly provided by Sharon Brookes, Animal Plant and Health Agency, Weybridge, United Kingdom.

### Minigenome assay.

eHAP dKO cells were transfected in 24-well plates using Lipofectamine 3000 (Thermo Fisher) with a mixture of plasmids: 100 ng of pCAGGs ANP32/pCAGGs empty, 40 ng of pCAGGs PB2, 40 ng of pCAGGs PB1, 20 ng of pCAGGs PA, 80 ng of pCAGGs NP, 40 ng of pCAGGs *Renilla* luciferase, and 40 ng of polI vRNA firefly luciferase. Transfections in wild-type eHap cells were performed similarly but without ANP32. Transfections in NPTr cells were carried out in 12-well plates using the same ratios as above. Twenty-four hours posttransfection, cells were lysed with passive lysis buffer (Promega) and luciferase bioluminescent signals were read on a FLUOstar Omega plate reader (BMG Labtech) using the Dual-Luciferase Reporter assay system (Promega). Firefly signal was divided by *Renilla* signal to give relative luminescence units (RLU). All assays were performed with 2 or 3 separate repeats on different days; representative experiments are shown.

### Virus replication assays.

All virus replication assays were performed with recombinant viruses containing the HA, NA, and M genes of A/Puerto Rico/8/1934 (H1N1; PR8) and the remaining genes from the avian influenza virus 50-92 containing PB2 627 E (wild type) or K, as has been described previously (11). eHAP dKO cells pretransfected 24 h prior with 400 ng of pCAGGs-ANP32A (chicken, swine, or human), pCAGGs-empty, or wild-type eHAP or NPTr cells were infected at a multiplicity of infection of 0.001 in 6-well plates. Virus growth media, either IMDM or DMEM (for eHAP cells and NPTr cells, respectively) was made from serum-free media containing 1 μg/ml of *N*-tosyl-l-phenylalanine chloromethyl ketone-treated trypsin (Worthington Biochemical). Virus-containing supernatants were collected at 12, 24, 48, and 72 h postinoculation and stored at −80°C. Titers were assessed by infectious plaques on MDCKs. All time points were taken in triplicate, and all virus growth curves were performed at least twice with a representative repeat shown.

### Split-luciferase assay.

Split-luciferase assays were undertaken in 293Ts seeded in 24-well plates. We cotransfected 30 ng each of PB2, PA, and PB1, with the N terminus of *Gaussia* luciferase (Gluc1) tagged to its C terminus after a GGSGG linker cotransfected using Lipofectamine 3000 along with ANP32A, tagged with the C terminus of *Gaussia* luciferase (Gluc2) on its C terminus (after a GGSGG linker). Twenty-four hours later, cells were lysed in 100 μl of *Renilla* lysis buffer (Promega), and *Gaussia* activity was measured using a *Renilla* luciferase kit (Promega) on a FLUOstar Omega plate reader. Normalized luminescence ratios (NLRs) were calculated by dividing the values of the tagged PB1 and ANP32 wells by the sum of the control wells, which contained (i) untagged PB1 and free Gluc1, and (ii) untagged ANP32A and free Gluc2 as described elsewhere (15, 37).

### Western blotting.

To confirm equivalent protein expressing during minigenome assays, transfected cells were lysed in radioimmunoprecipitation assay (RIPA) buffer (150 mM NaCl, 1% NP-40, 0.5% sodium deoxycholate, 0.1% SDS, 50 mM Tris, pH 7.4) supplemented with an EDTA-free protease inhibitor cocktail tablet (Roche).

Membranes were probed with mouse α-FLAG (catalog no. F1804; Sigma), rabbit α-Vinculin (catalog no. ab129002; Abcam), rabbit α-PB2 (catalog no. GTX125926; GeneTex), and mouse α-NP ([C43]; catalog no. ab128193; Abcam). The following near-infrared (NIR) fluorescent secondary antibodies were used: IRDye 680RD goat anti-rabbit (IgG) secondary antibody (catalog no. ab216777; Abcam) and IRDye 800CW goat anti-mouse (IgG) secondary antibody (catalog no. ab216772; Abcam). Western blots were visualized using an Odyssey imaging system (Li-Cor Biosciences).

### Immunofluorescence.

For investigating localization of exogenously expressed ANP32 proteins, eHAP ANP32 dKO cells were cultured on 8-well chambered coverslips (Ibidi) and transfected with 125 ng of the indicated FLAG-tagged ANP32 protein. Cells were fixed in PBS and 4% paraformaldehyde 24 h posttransfection and then permeabilized in PBS and 0.2% Triton X-100. Cells were blocked in PBS, 2% bovine serum albumin, and 0.1% Tween. FLAG-tagged ANP32 proteins were detected using mouse anti-FLAG M2 primary antibody (Sigma), followed by goat anti-mouse Alexa Fluor 568 (Invitrogen). Nuclei were counterstained with 4′,6-diamidino-2-phenylindole (DAPI). Images were obtained using a Zeiss Cell Observer widefield microscope with ZEN Blue software, using a Plan-Apochromat 63 × 1.40-numerical aperture oil objective (Zeiss) and processed using Fiji software (38).

For investigating endogenous levels of ANP32A in swine cells, NPTr cells were cultured in Nunc 24-well tissue culture plates on coverslips (VWR) preincubated with 10% (vol/vol) collagen (rat tail; Sigma-Aldrich) in PBS. Cells were fixed with PBS and 4% paraformaldehyde for 20 min at room temperature. Cells were permeabilized with PBS and 1% Triton X-100 for 10 min, followed by 3 washes with PBS and 0.1% Triton X-100 and blocking with PBS and 5% (wt/vol) skim milk powder for 1 h at room temperature. ANP32A was detected using anti-PHAP1 antibody (catalog no. ab189110; Abcam) incubated in PBS and 5% (wt/vol) skim milk powder overnight at 4°C, followed by incubation with anti-rabbit Alexa Fluor 488 (catalog no. ab150077; Abcam). Phalloidin was detected using an Alexa Fluor 647 conjugated antibody (catalog no. ab176759; Abcam), incubated during the secondary antibody application step at 1:10,000 concentration. Nuclei were counterstained with DAPI (1:15,000; Thermo Fisher). Images were captured with a Leica DMLB fluorescence microscope using Micro-Manager software at 40× and 20× for DAPI and phalloidin, respectively. Images were processed using Fiji software (38).
